# A case report of Ebstein’s anomaly–Gerbode defect dyad: is there room for another anomaly in the same patient?

**DOI:** 10.1093/ehjcr/ytae444

**Published:** 2024-08-22

**Authors:** Horea-Laurentiu Onea, Florin-Leontin Lazar, Minodora Teodoru, Oana Stoia, Dan-Mircea Olinic

**Affiliations:** Department of Internal Medicine, Iuliu Hatieganu University of Medicine and Pharmacy, 5th Dept. of Internal Medicine, Medical Clinic No.1, 40006 Cluj-Napoca, Romania; Department of Cardiology, County Clinical Emergency Hospital Sibiu, 550245 Sibiu, Romania; Department of Internal Medicine, Iuliu Hatieganu University of Medicine and Pharmacy, 5th Dept. of Internal Medicine, Medical Clinic No.1, 40006 Cluj-Napoca, Romania; Department of Cardiology, County Clinical Emergency Hospital Sibiu, 550245 Sibiu, Romania; Department of Cardiology, County Clinical Emergency Hospital Sibiu, 550245 Sibiu, Romania; Medical Clinical Department, Faculty of Medicine, Lucian Blaga University, 550024 Sibiu, Romania; Department of Cardiology, County Clinical Emergency Hospital Sibiu, 550245 Sibiu, Romania; Medical Clinical Department, Faculty of Medicine, Lucian Blaga University, 550024 Sibiu, Romania; Department of Internal Medicine, Iuliu Hatieganu University of Medicine and Pharmacy, 5th Dept. of Internal Medicine, Medical Clinic No.1, 40006 Cluj-Napoca, Romania; Second Cardiology Department, County Clinical Emergency Hospital Cluj-Napoca, 400006 Cluj-Napoca, Romania

**Keywords:** Case report, Congenital heart disease, Ebstein’s anomaly, Gerbode defect

## Abstract

**Background:**

Ebstein’s anomaly is a rare congenital heart disease characterized by apical displacement of the septal and posterior tricuspid valve leaflets. It is commonly associated with other defects such as patent foramen ovale or accessory atrioventricular pathways.

**Case summary:**

We describe a case of an Ebstein anomaly diagnosed in an adult in his 50s in association with a septal defect between the left ventricle and right atrium (Gerbode defect). The diagnosis was confirmed on magnetic resonance imaging. A third anomaly was noted on coronary angiography, consisting of an aberrant origin of the right coronary artery from the left sinus of Valsalva. The patient was paucisymptomatic until he developed typical atrial flutter. Catheter ablation was employed after first arrhythmia recurrence and the patient is to date in good clinical condition.

**Conclusion:**

The association of Ebstein’s anomaly–Gerbode defect is extremely rare, and to our knowledge, this is the first case that presents in addition an anomalous coronary artery. Both structural defects were without haemodynamic significance, and there was no proof of myocardial ischaemia. As the case illustrates, congenital disorders, even when in conjunction, can have a silent clinical course and multimodality imaging is sometimes necessary for a complete and final diagnosis.

**Learning points**

- In patients with structural heart defects screening for associated anomalies is required, as two or more entities can coexist in the same patient.
- Associated congenital heart defects can sometimes have a silent clinical evolution.
- Even when cardiac congenital defects are associated, a conservative management can be employed if the patient is asymptomatic.

## Introduction

With a reported incidence of 1 per 200 000 births, Ebstein’s anomaly is considered a rare disorder responsible for <1% of all congenital heart disease.^1^ The primary pathological feature consists of apical displacement and adherence to the underlying myocardium of the septal and posterior tricuspid valve (TV) leaflets in varying degrees.^2^ Common concomitant associated lesions in patients with Ebstein anomaly include patent foramen ovale/atrial septal defects and accessory atrioventricular pathways.^3^

The Gerbode defect represents a particular type of ventricular septal defect (VSD) consisting of a communication between the left ventricle (LV) and right atrium (RA), which in its congenital form accounts for only 0.08% of all intracardiac shunts.^4^ In this article, we describe a case of Ebstein’s anomaly and Gerbode defect, a very rare clinical association. To our knowledge, this is only the fourth reported case of an Ebstein–Gerbode dyad and the first case that presents an anomalous coronary artery, as the patient had a right coronary artery (RCA) originating from the left sinus of Valsalva.

## Summary figure

**Figure:**
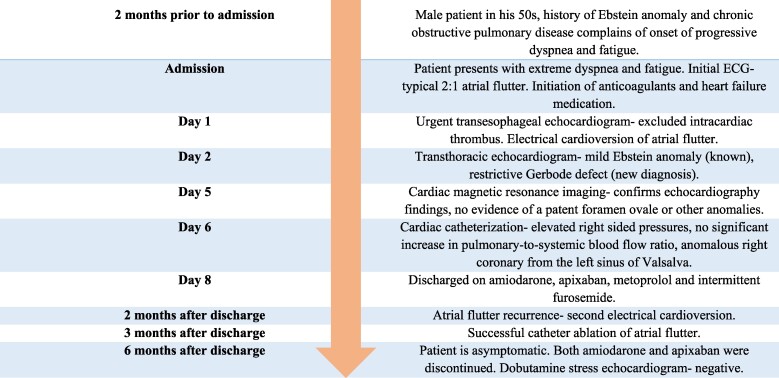


## Case presentation

A male patient in his 50s with a history of Ebstein’s anomaly and no documentation regarding his condition, chronic obstructive pulmonary disease, was admitted to the hospital complaining of extreme fatigue and exertional dyspnoea for the past 2 weeks. He was in NYHA functional class I, 2 months prior. His baseline medication included angiotensin-converting enzyme inhibitors and bronchodilators.

A pulmonary function test from 3 months prior shows mixed ventilatory pattern, with moderate restriction, moderate central obstruction, and severe peripheral obstruction. There is reduction of maximum ventilation by 55%.

On physical examination during the current admission, the spontaneous oxygen saturation was 88%, while the patient was haemodynamically stable. There was presence of a grade IV/VI systolic murmur at the left lower sternal border, pulmonary rhonchi, perioral cyanosis, clubbing and stasis dermatitis with 2 + pitting lower limb oedema.

**Figure 1:**
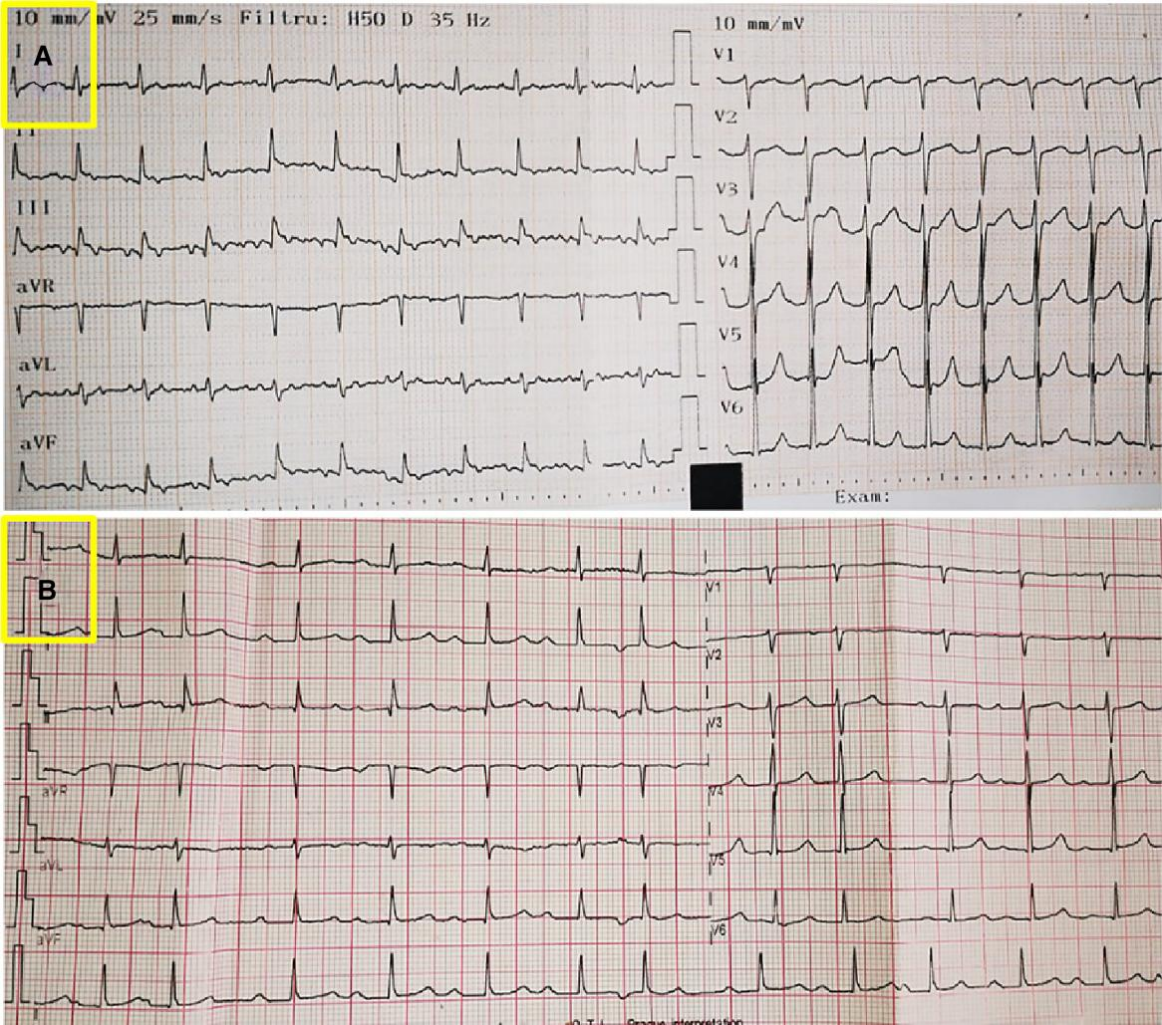
Electrocardiogram showing typical 2:1 atrial fibrillation at presentation (*A*) and sinus rhythm with first-degree atrioventricular block after electrical cardioversion (*B*). AF, atrial flutter. AV, atrioventricular; ECG, electrocardiography; SR, sinus rhythm.

**Figure 2:**
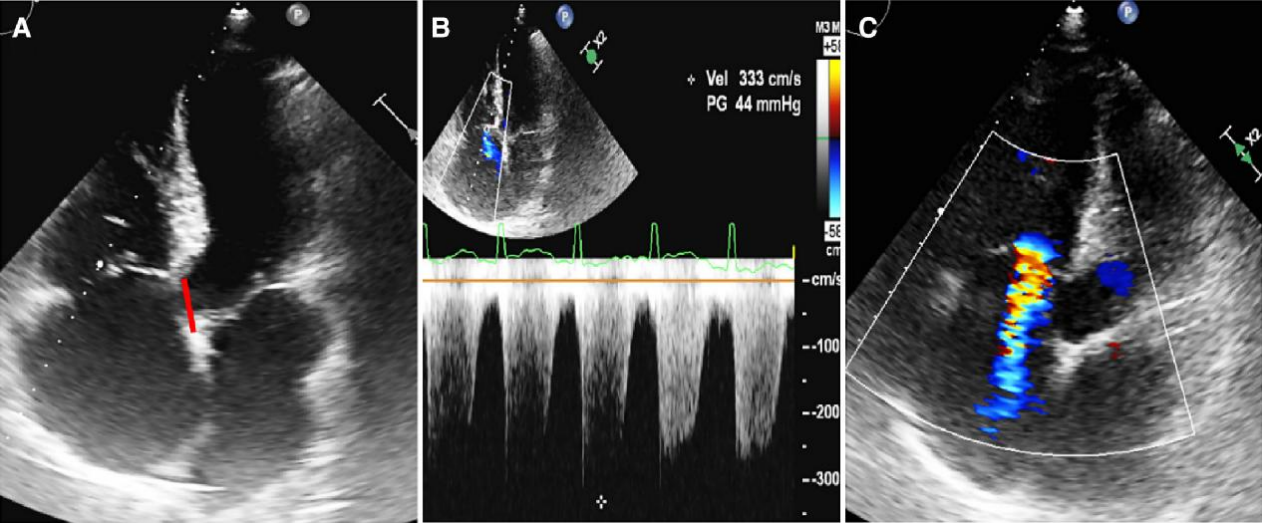
(*A*) Apical 4-chamber view revealing apical displacement of septal leaflet of the tricuspid (straight line) with (*B*) moderate TR. (*C*) Colour Doppler exhibiting tricuspid regurgitation with a jet velocity of 3.33 m/s and a maximum gradient of 44 mmHg. A4C, apical 4 chamber; TR, tricuspid regurgitation.

**Figure 3:**
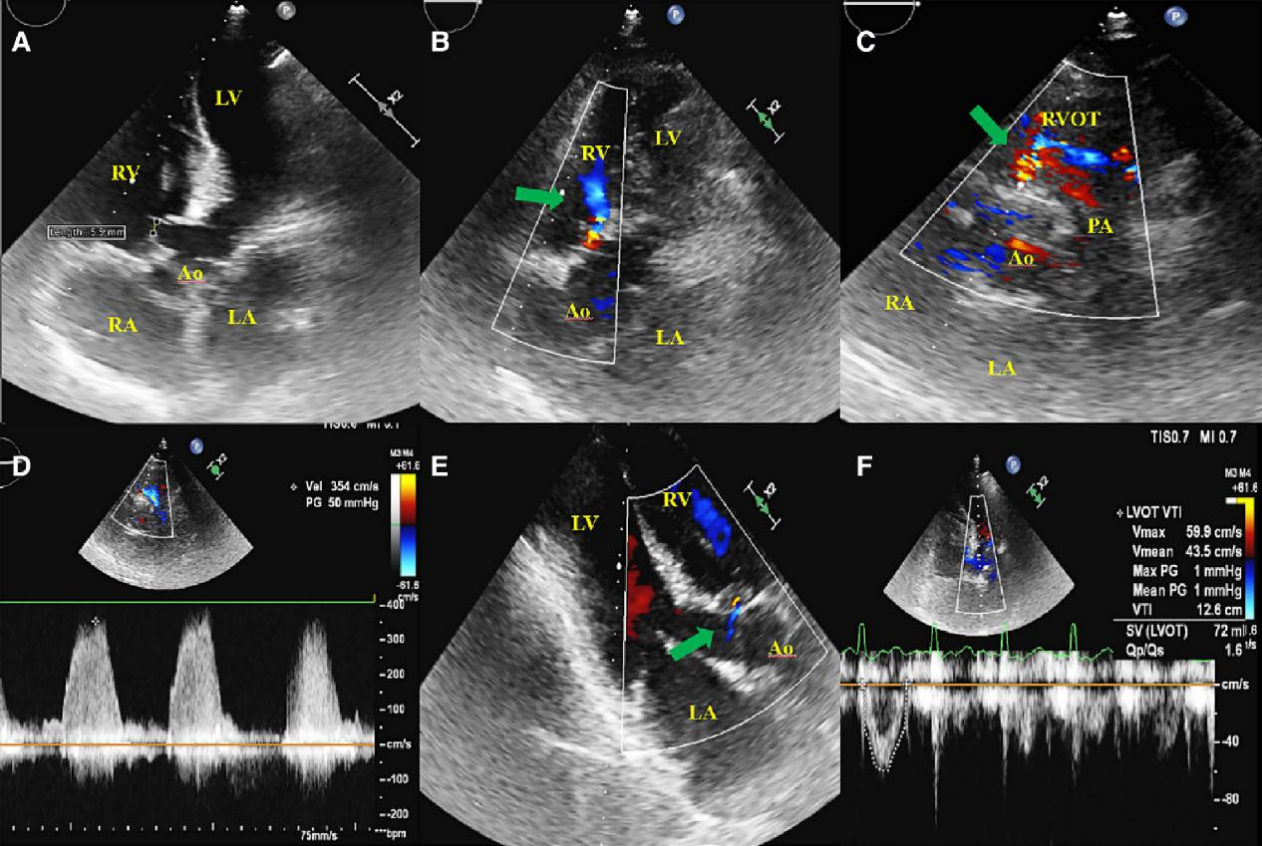
(*A*) Apical 5-chamber view showing a small restrictive perimembranous ventricular septal defect (5.5 mm) with predominantly (*B*) left-to-right shunting (arrow) on modified apical 5-chamber view. (*C*) Parasternal short-axis level of great vessels confirming the communication (arrow) with a (*D*) maximum gradient of 50 mmHg. (*E*) Parasternal long axis view with proof of right-to-left shunting (arrow). (*F*) Calculation of pulmonary-to-systemic blood flow ratio—1.6. A5C, apical 5 chamber; Ao, aorta; LA, left atrium; LV, left ventricle; PA, pulmonary artery; PLAX, parasternal long axis; PSAX, parasternal short axis; RA, right atrium; RV, right ventricle; RVOT, right ventricular outflow tract; VSD, ventricular septal defect.

Natriuretic peptides were mildly elevated (NT-proBNP 1679 pg/mL), whereas other blood tests were unremarkable. Initial electrocardiography (ECG) showed typical 2:1 atrial flutter (AF) (*Figure 1*). After exclusion of intracardiac thrombosis, external electrical cardioversion was performed (100 J), with conversion into sinus rhythm with first degree atrio-ventricular block and no ST-T changes (*Figure 1*). Low oxygen saturation persisted after cardioversion. Transthoracic echocardiogram confirmed the Ebstein’s anomaly (*Figure 2*; Supplementary material online, *Video S1*) corresponding to type A according to the Carpentier classification, indicative of a mild form, resulting in a moderate tricuspid regurgitation (*Figure 2*). Jet velocity was 3.33 m/s (gradient 44 mmHg; *Figure 2*), which translated into a high probability of pulmonary hypertension. Qualitatively, the right ventricle (RV) was moderately dysfunctional (TAPSE 14 mm) and mildly dilated. Left ventricular ejection fraction was ∼50%. A small restrictive peri-membranous VSD (gradient 50 mmHg) with predominantly left-to-right shunt (see Supplementary material online, *Videos S2* and *S3*) was present together with signs of pulmonary over-circulation (pulmonary-to-systemic blood flow ratio of 1.6; *Figure 3*). There was no evidence of a patent foramen ovale/atrial septal defect.

**Figure 4:**
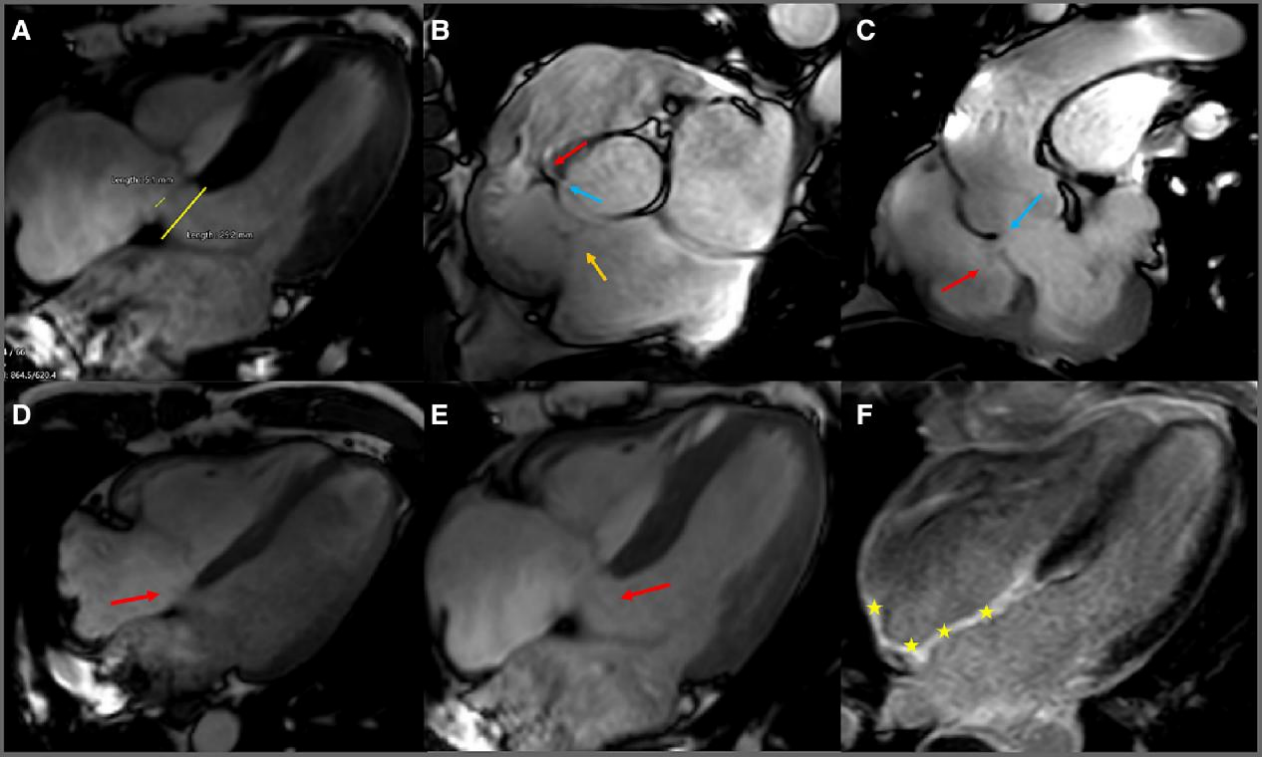
(*A*) Contrast magnetic resonance imaging showing apical displacement of septal leaflet of the tricuspid by 12.5 mm/m^2^—Ebstein’s anomaly (broad line)—and a 5.1 mm communication (thin line) between the LV and the RA, Gerbode defect. (*B*) Modified short-axis cine of the aortic valve—TV (yellow arrow), defect (blue arrow), flow acceleration (red arrow). (*C*) Cine left ventricular outflow tract sequence showing the defect (blue arrow) and the flow acceleration (red arrow)—left-to-right shunt. (*D*) Left-to-right shunt (arrow). (*E*) Right-to-left shunt (arrow). (*F*) LGE at the level of the ight atrium (star). CMR, contrast magnetic resonance; LGE, late gadolinium enhancement; LV, left ventricle; LVOT, left ventricular outflow tract; RA, right atrium; RV, right ventricle; TV, tricuspid valve.

In order to provide a better characterization of the above (as the echography window was occasionally suboptimal) and exclude other possible associated abnormalities, contrast magnetic resonance imaging was employed. Septal leaflet of the tricuspid valve (TV) was displaced apically by 25.2 mm (12.5 mm/m^2^) with minimal atrialization of the RV (*Figure 4*, Supplementary material online, *Video S4*). The membranous interventricular septum exhibits an aneurysmal segment, 11.5 mm in length, together with a 5.1 mm solution of continuity between the LV and the RA (consistent with a Gerbode defect; *Figure 4*; Supplementary material online, *Video S5*). There was noticeable bidirectional shunting (*Figure 4*; Supplementary material online, *Videos S4–SS6*). Magnetic resonance imaging showed marked dilation of the RA, while the RV was mildly dilated (end-diastolic volume 133 mL/m^2^) with a decreased ejection fraction of 32% (*Table 1*). Pulmonary valve anatomy was normal, and all four pulmonary veins drained into the left atrium (LA). On late gadolinium enhancement (LGE) imaging, there was a non-negligible degree of RA wall fibrosis (*Figure 4*).

**Table 1:** Cardiac magnetic resonance imaging relevant parameters

LV EDV, ml (mL/m^2^)	170.3 (84.2)
LV ESV, ml (mL/m^2^)	81.8 (40.5)
LV EF, %	52
RV EDV, ml (mL/m^2^)	268.7 (133)
RV ESV, ml (mL/m^2^)	182.7 (90.4)
RV EF, %	32
LA max volume^a^	43 (86.9)
RA max volume^a^	112 (55.4)
Qp:Qs^b^	1.5

EDV, end-diastolic volume; EF, ejection fraction; ESV, end-systolic volume; LA, left atrium; LV, left ventricle; Qp:Qs, pulmonary-to-systemic blood flow ratio; RA, right atrium; RV, right ventricle.

^a^Calculated using biplane area-length method.

^b^Calculated through phase contrast sequence.

**Figure 5:**
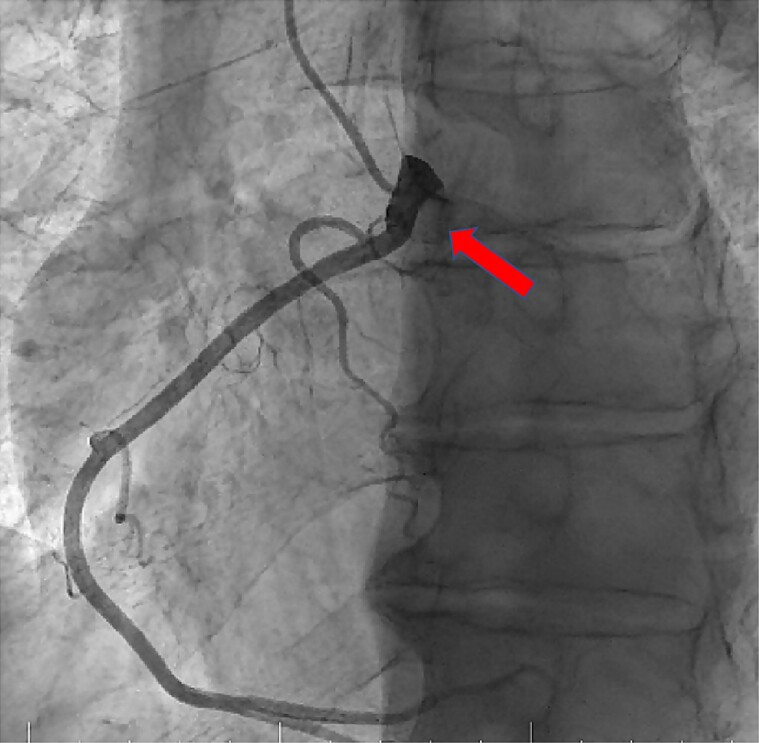
Coronary angiography showing anomalous origin of the right coronary artery (arrow) from the left sinus of Valsalva and no significant lesions.

Cardiac catheterization was performed in order to assess the haemodynamic status and presence of coronary artery disease. Right-sided pressures were elevated, translating into a mean RA pressure of 21 mmHg, an RV pressure of 54/13 mmHg, and mean pulmonary artery pressure of 32 mmHg. Calculation of the pulmonary-to-systemic blood flow ratio (Fick method) returned a borderline value of 1.5. Oximetry measurements were notable for a step up between the superior vena cava and RA (oxygen saturation 78%) and step down at the level of the LV–aorta (Ao) site (oxygen saturation 85%). Coronary angiography revealed non-obstructive coronary artery disease and an anomalous origin of the RCA from the left sinus of Valsalva (*Figure 5*).

The patient had an uneventful in-hospital evolution and was discharged following volume depletion. Chronic at-home treatment included class III anti-arrhythmics, direct anticoagulants, beta-blockers, intermittent diuretics, and bronchodilators. Two months later, he suffered a recurrence of AF and was thus scheduled for catheter ablation, without any peri-procedural complications. At his last outpatient visit (3 months after ablation), he was in good clinical condition and paucisymptomatic, and both anti-arrhythmics and anticoagulants (CHA2DS2-VASc score = 1) were discontinued. A decision was made to perform a dobutamine stress echocardiogram given his anomalous RCA, which was negative for angina symptoms, life-threatening arrhythmias, or wall motion abnormalities.

## Discussion

Ebstein’s anomaly is a rare congenital heart disease primarily involving the TV and the adjacent RV myocardium. Antero-caudal displacement of the TV (of at least 8 mm/m^2^ body surface area) and ‘atrialization’ of the RV is responsible for RV dysfunction and tricuspid regurgitation of varying degrees.^2^

Depending on the severity of valve displacement, a wide spectrum of clinical manifestations can occur. Mild forms of the disease may be asymptomatic, or diagnosed in the adulthood through an incidental murmur, arrhythmias, or right HF failure symptoms. Conversely, severe forms can present at any age with cyanosis, arrhythmias, and HF, while the most severe cases present as neonates.^5^

An interatrial communication is a frequent concomitant finding in these patients, resulting in a right-to-left shunt and various degrees of cyanosis.^3^ Other associated lesions include electrophysiological abnormalities (accessory atrioventricular pathways most commonly), mitral valve prolapse, bicuspid aortic valve, pulmonary atresia, or LV noncompaction.^3^ An isolated VSD is a rare association (8% in a cohort of 106 patients).^6^

Clinical symptoms usually dictate further management. Asymptomatic patients can safely be managed conservatively. According to the latest European Guidelines on the management of adult congenital heart disease,^7^ surgical intervention is recommended in symptomatic patients with severe tricuspid regurgitation or in case of progressive RV dilation or dysfunction when a tricuspid repair is feasible. Symptomatic arrhythmias are preferably treated with electrophysiologic intervention.^7^

Gerbode defect represents the least common type of VSD involving the membranous part of the interventricular septum.^4^ While acquired defects are increasingly being reported, congenital cases are rare. Since the membranous septum has a slight apical position relative to the mitral annulus, it is divided by the septal leaflet of the TV into an atrioventricular and interventricular segment.^8^ Three types have been contemporary described, but in a true Gerbode defect (supravalvular type), there is a communication between the LV and RA at the level of the atrioventricular septum.^8^

The occurrence of Ebstein’s anomaly and Gerbode defect in the same patient is very uncommon, with only three case reports found in the literature,^9,10,11^ two of which in an adult patient. In the first case,^9^ a more severe form of Ebstein’s anomaly was observed (37 vs. 25 mm TV displacement in our case) thus explaining his worse clinical status. Our patient was paucisymptomatic until he developed AF. Interestingly, in the former, the patient was cyanotic due to the right-to-left shunt via a persistent foramen ovale, in contrast to our case who did not develop this shunt and was most likely cyanotic due to his associated lung disease. Nevertheless, intermittent right-to-left shunt was observed in our patient, probably due to right-sided pressure elevation related to voluntary Valsalva manoeuver during imaging evaluation. The case by Bayar *et al.* documented another form of atrial arrhythmia, the Wolff–Parkinson–White syndrome that was treated exclusively medically.^10^

Our patient was referred for catheter ablation of symptomatic AF, which was successful. However, given the degree of RA fibrosis on LGE, occurrence of additional atrial arrhythmias might be expected. No further invasive treatment was deemed necessary at the moment, as the patient did not present any symptoms related to the structural defects. What is more, the Gerbode defect was restrictive, with no significant increase in cardiac flow ratio and in consequence, right-sided chamber enlargement was attributed to his associated pathology. The elevated mean pulmonary pressure was most likely due to a combination of pre- and post-capillary aetiology.

Aberrant origin of the RCA from the left sinus of Valsalva is an extremely rare form of coronary anomaly that can have either a benign (intraseptal, prepulmonic, or retroaortic) or malignant (interarterial) course based on the risk of sudden cardiac death. Latest European Guidelines^7^ recommend a conservative management in asymptomatic patients without ischaemia or high-risk anatomical features. Given that our patient survived to this age, the lack of typical angina symptoms and the negative stress test strongly suggest a benign RCA course.

We described a case of a male adult in which the rare association of a mild form of Ebstein’s anomaly and a restrictive Gerbode defect had an uneventful and asymptomatic clinical evolution until the development of a supraventricular tachycardia. Particular to our case was the presence of moderate/severe pulmonary disease and the association of another rare anomaly, consisting in an aberrant origin of the RCA from the left coronary sinus, however, without any clinical or haemodynamic significance. Following catheter intervention, the patient’s evolution was to date eventless, without arrhythmia recurrence.

Our case emphasizes the need for meticulous investigation through multimodality imaging in order to facilitate detection of associated congenital defects in the same patient.

## Lead author biography


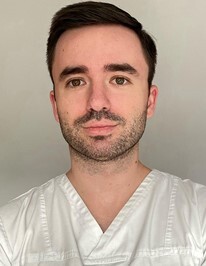


Horea Laurentiu Onea graduated from the University of Medicine and Pharmacy Iuliu Hatieganu of Cluj-Napoca in 2016. He is a general cardiologist, PhD(c), and fellow in interventional cardiology at the County Clinical Emergency Hospital of Cluj-Napoca. His main areas of interest include multimodality cardiac imaging, percutaneous coronary interventions, and intravascular imaging.

## Supplementary Material

ytae444_Supplementary_Data

## Data Availability

The data underlying this article will be shared on reasonable request to the corresponding author.

## Supplementary material

**Consent:** The patient has provided written consent for publication, in accordance with the COPE guidelines.

**Funding:** This work was funded by the Ministry of Research, Innovation, and Digitization through Program 1—Development of the national research-development system, Subprogram 1.2—Institutional performance- Projects for financing excellence in RDI, contract no. 28PFE/30.12.2021.
